# Open Repair of a Large Abdominal Aortic Aneurysm With a Type 2 Endoleak After an Endovascular Aortic Aneurysm Repair: A Case Report and Literature Review

**DOI:** 10.7759/cureus.40315

**Published:** 2023-06-12

**Authors:** Eman H Elbayoumi, Houssam Farres, Young Erben

**Affiliations:** 1 Vascular and Endovascular Surgery, Mayo Clinic, Jacksonville, USA

**Keywords:** endoleak repair, abdominal aortic aneurysm, endovascular aneurysm repair, type 2 endoleak, open repair

## Abstract

A type 2 endoleak (T2E) can occur after an endovascular aortic aneurysm repair (EVAR). The repair of a T2E is recommended after a sac enlargement of ≥5mm. We present a unique case of a 10 cm aneurysm sac that underwent open explantation 11 years after the initial EVAR and after having undergone several interventions to address the T2E.

## Introduction

Endovascular aortic aneurysm repair (EVAR) is a widely used operative technique to address abdominal aortic aneurysm (AAA) [1]. Stent graft failure secondary to endoleaks, migration, endotension, and sac enlargement are persistent problems that can result in aneurysm rupture [2]. There are five different types of endoleaks including types 1 and 3, which can cause serious sudden problems including aortic rupture, and therefore, should be addressed immediately when found [3,4]. Type 4 and 5 endoleaks are extremely rare with the new generations of endograft for AAA repair [5]. A type 2 endoleak (T2E) tends to have a more benign natural history; however, if it does not resolve spontaneously and the AAA sac enlarges, the side branch vessel feeders should be embolized to prevent further sac growth [6]. Several methods to treat these endoleaks are currently accepted among interventionalists, including transcaval and/or transarterial embolization using a combination of glue and coils. Another approach is direct aneurysm sac puncture via fluoroscopic and/or CT guidance [7]. Herein, we report a case of an enlarging AAA of 10cm in diameter without rupture with several prior interventions to stop aneurysm sac growth as a result of a T2E. Due to failed interventions and continued growth of the aneurysm sac, an open surgical repair was performed with excision of the endograft and placement of a Rifampin-soaked 22 mm x 11 mm Dacron graft.

## Case presentation

**Figure 1:**
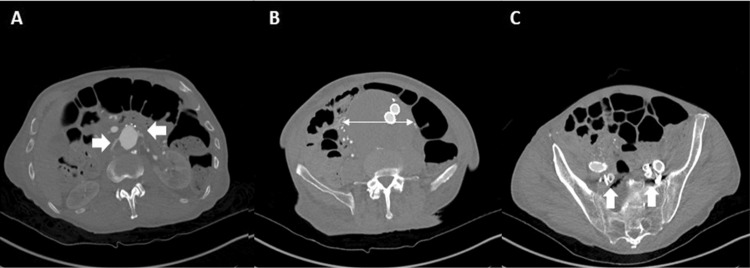
CTA of the patient on presentation A: Axial cut of CTA at the level of the right and left renal arteries (white arrows); B: Axial cut of CTA at the level of aneurysm sac's largest dimension (double white arrow); C: Axial cut of CTA at the level bilateral occluded internal iliac arteries (white arrows) CTA: Computed tomography angiography

A 78-year-old male patient with AAA was treated in 2012 with EVAR. His past medical history was relevant for small cell lung cancer (for which he underwent lobectomy followed by chemotherapy in 2015), emphysema, prostate cancer (treated with brachytherapy in 2018), hypertension, chronic obstructive pulmonary disease, and current history of tobacco use of one pack-per-day. It was noted that since his initial repair, the AAA sac continued to enlarge due to a presumed T2E. Per the patient history, he underwent three previous endovascular embolization procedures in 2015, 2018, and 2021, which included, bilateral internal iliac and lumbar arteries embolization in order to halt aortic sac growth. On current presentation, he complained of continued abdominal pain, which had been acutely present for several weeks. There were no other sources for his abdominal pain except the enlarging AAA sac measured to be 10cm in diameter on CT angiography (CTA) as seen in Figure 1.

**Figure 2:**
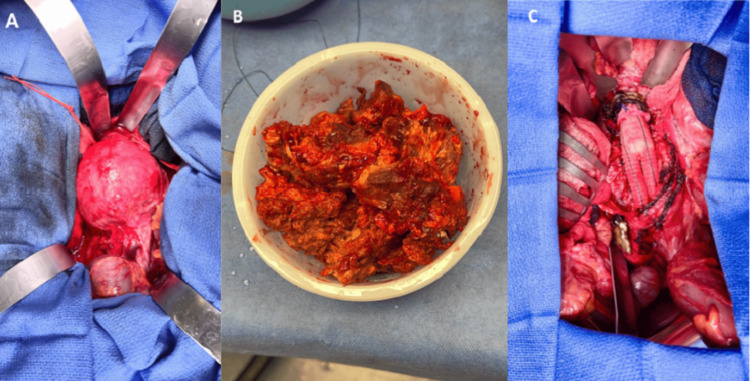
Intraoperative images A: Exposure of the 10 cm aneurysm; B: Aneurysm thrombus removed; C: Image of the 22 mm x 11 mm Rifampin-soaked Dacron graft replacing A

Appropriate multi-disciplinary discussions occurred to optimize this patient for open surgery including cardiology, pulmonology, nutrition, and critical care, among others. After weighing the risks vs benefits of explantation, he then underwent open endograft excision through a transperitoneal approach. A suprarenal clamp was initially placed to facilitate the proximal anastomosis, which was then moved to an infrarenal location prior to performing the distal anastomosis. A 22 mm x 11 mm Rifampin-soaked Dacron graft was utilized for the repair (Figure 2). The distal anastomoses were performed with partial resection of the iliac limbs since they could not be easily removed.

**Figure 3:**
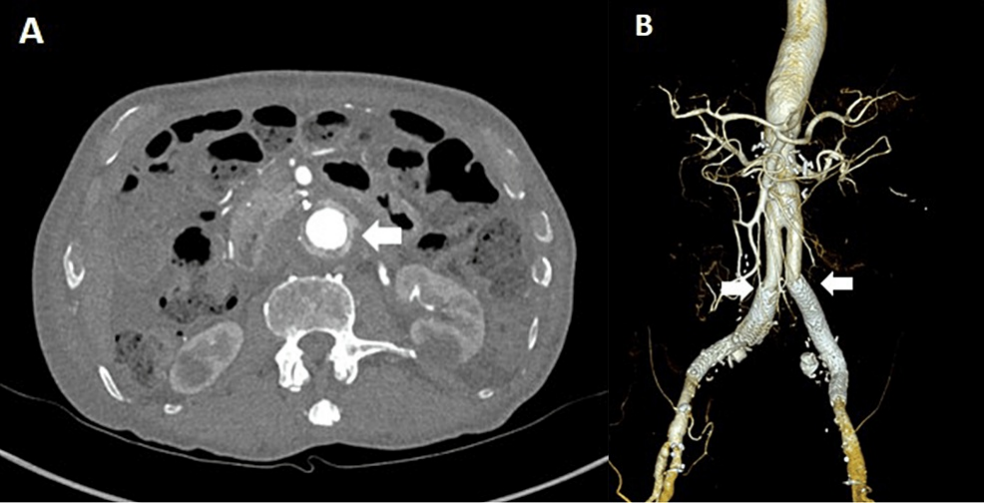
CTA on postoperative day 42 A: Axial cut at the level of proximal infrarenal anastomosis depicting felt strip at the anastomosis (white arrow); B: Three-dimensional reconstruction of the EVAR explantation depicting the distal anastomosis to the bilateral iliac limbs (white arrows) CTA: Computed tomography angiography, EVAR: Endovascular aortic aneurysm repair

Postoperatively, the patient recovered well, he regained bowel function on postoperative day 2 and was discharged from the hospital on postoperative day 6. He was followed up with two phone calls until his return for a postoperative visit on postoperative day 42 when the CTA demonstrated an excellent repair with no abdominal pain (Figure 3).

## Discussion

**Figure 4:**
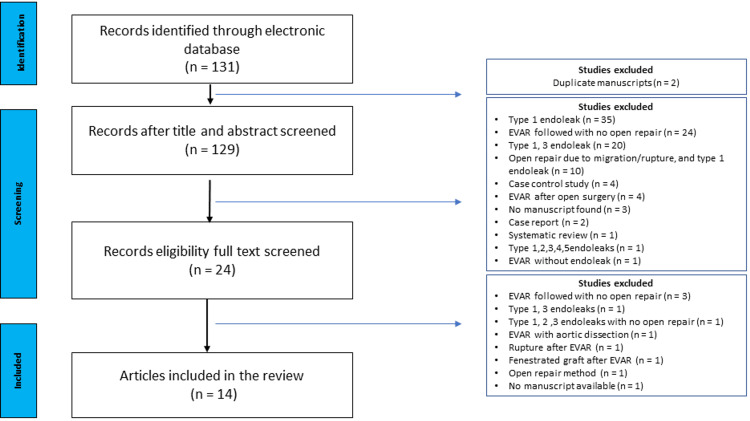
PRISMA flow diagram of open repair after EVAR and T2E RISMA: Preferred Reporting Items for Systematic Reviews and Meta-Analyses, EVAR: Endovascular aortic aneurysm repair, T2E: Type 2 endoleak

The aim of AAA repair by either surgical or endovascular techniques is to reduce the risk of rupture and death [8]. Through the development of EVAR, we can perform this safely and with improved morbidity and mortality compared to traditional open repair [9]. Endoleaks seem to be the Achilles' heel of EVAR, which can cause aneurysm sac growth and ultimately rupture [10]. So, elective open explantation of the EVAR should be considered when all possible endovascular options have been exhausted [11]. Ours is a unique case of aneurysm sac growth to 10cm in diameter without rupture over an 11-year period after initial EVAR. Several attempts were made unsuccessfully to address the T2E. The large aneurysm was then repaired using an open surgical approach. We performed a literature review of all cases undergoing open repair after EVAR due to a persistent T2E. This literature review was initially composed of 131 manuscripts; out of which 14 were selected for our in-depth analysis. A detailed selection criteria is shown in Figure 4. There were 10 retrospective cohort studies and four prospective reviews published between 2000 and 2023. Follow-up ranged between one and five years. There were 180 open explantations out of a total of 8097 EVARs (Table 1). The size of the aortic sac varied between 5.39 cm and 8.0cm in diameter and at least 570 patients needed at least one endovascular reintervention to address the T2E prior to the open repair.

**Table 1:** A literature review on open repair after T2E following EVAR T2E: Type 2 endoleak, EVAR: Endovascular aortic aneurysm repair

Year of publication	Author	Type of study	Number of patients	Number of T2E	Patients needing one reintervention after EVAR to address T2E	Long term outcome (death due to T2E, persistent endoleak, rupture due to T2E)	Mean and/or median of sac diameter growth	Open repair
2022	Le et al. [12]	Retrospective cohort Study	3891	132	119	-	Mean=5.7 ± 1.4 cm	26
2021	Chastant et al. [13]	Retrospective cohort	62	28	14	-	Mean=7.13 ± 2.19 cm	62
2021	Bacquemin et al. [14]	Prospective cohort multicenter study	180	6	4	1 death	Mean=5.66 ± 0.85 cm	1
2020	Midy et al. [15]	Prospective cohort multicenter study	176	40	-	1 persistent endoleak	Mean=5.39 ± 0.86 cm	2
2019	Law et al. [16]	Retrospective cohort study	667	6	3	-	Mean=8.0 ± 1.8 cm	22
2019	Teijink et al. [17]	Retrospective cohort study	1263	265	253	25 deaths	Mean > 7 cm	26
2018	Bastiaenen et al. [18]	Retrospective case series study	11	2	-	1 persistent endoleak	Median=5.2-7.8 cm	1
2018	Massara et al. [19]	Retrospective cohort study	150	12	8	1 persistent endoleak	Median=5.86 cm	1
2017	Perini et al. [20]	Retrospective descriptive study	28	4	4	4 persistent endoleaks	-	4
2014	Klonaris et al. [21]	Retrospective cohort study.	442	10	7	10 persistent endoleaks	-	10
2014	Botsios et al. [22]	Retrospective cohort study	411	2	-	1 persistent endoleak 1 rupture related to endoleak	-	9
2013	El Batti et al. [23]	Prospective cohort study	700	200	49	88 persistent endoleaks	Mean=5.62 ± 0.89 cm	4
2012	Sarac et al. [24]	Retrospective cohort study	95	95	95	4 persistent endoleaks	-	8
2011	Brinsrer et al. [25]	Prospective cohort study	21	4	14	4 persistent endoleaks	-	4
Total			8,097	806	570	141	Mean=5.39-8.0 cm Median=5.2-7.8 cm	180

The Society for Vascular Surgery's recommendations for endoleak repair after EVAR is based on aneurysmal sac expansion of ≥5mm and the presence of symptoms [3]. Surveillance is key in the determination of timing for reintervention in the presence of a T2E. The recommended imaging protocol includes a CTA at one, six, and 12 months and yearly thereafter. In the absence of an endoleak and the presence of a stable sac size after one year, surveillance with ultrasound is a safe alternative option [3,26]. In the case of a T2E with ≥5mm sac growth, endovascular intervention can be performed using several different techniques including translumbar and/or transcaval embolization, using microcatheter techniques through the superior mesenteric artery branches/internal iliac artery branches, among others [27]. Late open conversion is sometimes necessary for continued aneurysm enlargement. It is debated when this open conversion should occur, as these procedures have a higher risk for complications. A study conducted by Chastant et al., including 62 patients, demonstrated that patients who underwent an elective late open conversion after EVAR had a survival rate of 58.8% and a reoperation rate of 12.9% at a mean follow-up of 28.4 months [13]. In a second study by Botsios et al., nine patients required late open conversions after initial EVAR (n=411). Around 81% survived this open procedure at a follow-up range of four to 60 months [22]. These studies highlight the range of significant postoperative risks of performing open explantation and also the significance of continued monitoring after EVAR [28]. Open surgical repair should be considered and performed if the aneurysm sac continues to grow and/or causes symptoms. One should consider the size threshold at which open explantation of the endograft should be performed.

## Conclusions

The approach to AAA care should be individualized. Although EVAR has decreased the morbidity and mortality of AAA pathology, it is key to surveil patients post-EVAR due to a few individual cases where an aneurysm sac continues to grow as a result of endoleaks. Endovascular techniques can address most endoleaks when the natural history of T2E is benign. However, an open surgical approach should remain an option in those patients whose aneurysm sacs continue to expand after failed minimally invasive techniques, as in our case.
